# Single-step bioconversion of lignocellulose to hydrogen using novel moderately thermophilic bacteria

**DOI:** 10.1186/1754-6834-7-82

**Published:** 2014-06-03

**Authors:** Guang-Li Cao, Lei Zhao, Ai-Jie Wang, Zhen-Yu Wang, Nan-Qi Ren

**Affiliations:** 1 School of Life Science and Technology, Harbin Institute of Technology, Harbin 150090, China; 2 State Key Laboratory of Urban Water Resource and Environment, Harbin Institute of Technology, Harbin 150090, China

**Keywords:** Lignocellulose, *Thermoanaerobacterium thermosaccharolyticum*, Biohydrogen, Degradation, Consolidated bioprocessing

## Abstract

**Background:**

Consolidated bioprocessing (CBP) of lignocellulosic biomass to hydrogen offers great potential for lower cost and higher efficiency compared to processes featuring dedicated cellulase production. Current studies on CBP-based hydrogen production mainly focus on using the thermophilic cellulolytic bacterium *Clostridium thermocellum* and the extremely thermophilic cellulolytic bacterium *Caldicellulosiruptor saccharolyticus*. However, no studies have demonstrated that the strains in the genus *Thermoanaerobacterium* could be used as the sole microorganism to accomplish both cellulose degradation and H_2_ generation.

**Results:**

We have specifically screened for moderately thermophilic cellulolytic bacteria enabling to produce hydrogen directly from conversion of lignocellulosic materials. Three new strains of thermophilic cellulolytic bacteria in the genus *Thermoanaerobacterium* growing at a temperature of 60°C were isolated. All of them grew well on various plant polymers including microcrystalline cellulose, filter paper, xylan, glucose, and xylose. In particular, the isolated bacterium, designated as *Thermoanaerobacterium thermosaccharolyticum* M18, showed high cellulolytic activity and a high yield of H_2_. When it was grown in 0.5% microcrystalline cellulose, approximately 82% cellulose was consumed, and the H_2_ yield and maximum production rate reached 10.86 mmol/g Avicel and 2.05 mmol/L/h, respectively. Natural lignocellulosic materials without any physicochemical or biological pretreatment also supported appreciable growth of strain M18, which resulted in 56.07% to 62.71% of insoluble cellulose and hemicellulose polymer degradation in corn cob, corn stalk, and wheat straw with a yield of 3.23 to 3.48 mmol H_2_/g substrate and an average production rate of 0.10 to 0.13 mmol H_2_/L/h.

**Conclusions:**

The newly isolated strain *T. thermosaccharolyticum* M18 displayed effective degradation of lignocellulose and produced large amounts of hydrogen. This is the first report of a *Thermoanaerobacterium* species presenting cellulolytic characteristics, and this species thus represents a novel cellulolytic bacterium distinguished from all other known cellulolytic bacteria. In comparison, the extraordinary yield and specific rate of hydrogen for strain M18 obtained from lignocellulose make it more attractive in monoculture fermentation. *T. thermosaccharolyticum* M18 is thus a potential candidate for rapid conversion of lignocellulose to biohydrogen in a single step.

## Introduction

Considering the detrimental effect of fossil fuel utilization on the environment and energy depletion, there is a pressing need to develop clean-burning and renewable energy that can replace fossil fuel derived energy, such as petroleum and coal. Hydrogen is recognized as an alternative future fuel to meet this demand because it is clean and renewable and has a high energy yield. Among the various hydrogen production technologies, anaerobic fermentative H_2_ production from organic wastes is considered to be an environmentally friendly and energy-saving biological process [1,2]. For this process to be economically competitive, renewable and low cost feedstock should be developed to provide a cost-effective energy supply [3].

Cellulosic biomass from agricultural, forest, and industrial residues is among the earth’s most abundant renewable natural resources and is an attractive, low-cost feedstock for biofuel production. Current strategies to produce biofuel from this feedstock mostly employ the processes of separate hydrolysis and fermentation (SHF) or simultaneous saccharification and fermentation (SSF), which require extensive pretreatment such as steam-explosion and acid treatment, followed by the addition of exogenous cellulase to hydrolyze cellulose and release reducing sugars for further fermentation [4-6]. However, during the hydrolysis step, large amounts of expensive commercial cellulases are usually needed, which increase the cost and hinder the commercialization of cellulosic biohydrogen production.

Consolidated bioprocessing (CBP), also called direct microbial conversion, featuring cellulase production, cellulose hydrolysis, and fermentation in one step, is widely recognized as the most attractive strategy for converting cellulosic biomass to biofuel, since it offers outstanding potential for lower costs and higher efficiency due to its simpler feedstock process, shorter time consumption, and lower energy input [7,8]. Researchers have pointed out that CBP has the potential to reduce costs more than 50% compared to other cellulase dedicated processes such as SSF or SHF [9]. CBP is, therefore, an economically attractive goal for biofuel production processes from lignocellulosic biomass including cellulosic hydrogen production. To realize this potential, the microorganisms involved in CBP must be able to solubilize a practical biomass substrate with high rate and high conversion, and simultaneously produce a desired product at high yield. Although many reported microorganisms possess the capability of cellulose hydrolysis or H_2_ production, until now, little research has clarified that both of these capabilities are possessed in a single microorganism. Compared with mesophiles, thermophiles are thought to be more robust for cellulose degradation and hydrogen production. In particular, the rate of cellulolysis is presumably more rapid at elevated temperatures [10,11]. As a result, thermophilic microorganisms isolated from various environments are an attractive prospect for cellulolytic biohydrogen production from complex lignocellulosic biomass. Nevertheless, previous studies on isolating cellulolytic bacteria have relied on selective enrichment in cellulose media, the diversity of the bacterial community in enrichment has not been monitored, and it has not been clear whether the strains enriched in liquid media have subsequently been successfully isolated on agar plates and obtained in pure culture. This method may thus miss novel isolates with the ability to degrade cellulose. Moreover, current studies on CBP-based hydrogen production mainly concentrate on using co-cultures of the thermophilic cellulolytic bacterium *Clostridium thermocellum* with non-cellulolytic thermophilic anaerobic bacteria and the extremely thermophilic cellulolytic bacterium *Caldicellulosiruptor saccharolyticus*[12,13]. No studies have demonstrated that the strains in the genus *Thermoanaerobacterium* could be used as the sole microorganism to accomplish both cellulose degradation and H_2_ production.

Here we present the results of screening for moderately thermophilic bacteria capable of producing H_2_ directly from lignocellulosic biomass. For determining whether the representative microorganisms present in the enrichment were successfully cultured, a community analysis during enrichment was investigated. We demonstrated that the newly isolated novel *Thermoanaerobacterium* strains were able to efficiently degrade various real lignocellulosic substrates for hydrogen production.

## Materials and methods

### Enrichment and isolation

Environmental samples were collected from rotted wood crumb, cow manure compost, and spring sludge. Enrichment cultures were carried out without shaking at 60°C in 100-mL top-sealed serum bottles with a working volume of 50 mL. Briefly, 1 g diluted (1:10) samples were incubated into the modified ATCC 1191 medium (MA), which consists of (per liter) 1.0 g NH_4_Cl, 3.0 g K_2_HPO_4_, 1.5 g KHB_2B_PO_4_, 0.5 g MgCl_2_∙6H_2_O, 1.0 g NaCl, 0.2 g KCl, 0.5 g cysteine-HCl, 2.0 g yeast extract, 5.0 g Avicel (PH-101, Fluka), 1 mL trace element solution, and l mL vitamin solution [14]. After five days of cultivation, the resultant culture broth was transferred at 10% (v/v) to fresh MA medium and cultured for another five days. This process was repeated several times successively in the same manner until the cultures had a stable microbial community (number and intensity of bands), which was examined by polymerase chain reaction (PCR)-denaturing gradient gel electrophoresis (DGGE) analysis (see below).

Once the dominant bacteria were determined based on the DGGE band sequence, we tried to isolate the bacterium directly from the last generation culture. Tenfold serial dilutions were plated on the solid MA medium (2%, w/v, agar) prepared in a tube and incubated at 60°C for five days. The agar samples containing well-formed cellulose-clearing colonies were transferred to fresh MA liquid medium under N_2_ gas flow and placed into an 85°C water bath for 5 min to liberate the cells from the agar. Repeated plating was done multiple times to ensure the purity of the isolated colonies. Further verification of purity was ensured by microscopy, colony morphology, and 16S rRNA gene sequencing. The capability to utilize 0.5% v/v cellulose by isolates was observed in batch tests. Isolates with high H_2_ production potential from cellulose were identified and tested.

### DGGE-PCR

The total community DNA was extracted from the enrichment cultures and purified using the bacterial DNA mini kit (Watson Biotechnologies Co. Ltd., Shanghai, China) according to manufacturer instructions. DGGE was performed according to the method described by Xing et al. [15]. The variable V3 region of 16S rDNA was amplified by PCR with the following primers: BSF338 (5′-ACTCCTACGGGAGGCAGCAG-3′, nt 338 to 354 by *E. coli* numbering), which was attached to a GC clamp (CGCCCGCCGCGCCCCGCGCCCGTCCCGCCGCCCCCGCCCG) at the 5′ terminus and primer BSR534 (5′-ATTACCGCGGCTGCTGG-3′, nt 517 to 534 by *E. coli* numbering). The PCR products then were separated by DGGE using a DCode universal mutation detection system (Bio-Rad Laboratories, Hercules, CA, USA). Prominent DGGE bands were extracted and purified from the gel bands. After reamplification under the reaction conditions described above, the resulting PCR products were cloned for sequencing.

### 16S rRNA gene sequencing

The genomic DNA of the isolated strains was extracted from the cellular precipitate of the culture broth using a DNA extraction kit as mentioned above. The extracted DNA was used as the template for PCR amplification of the 16S rRNA gene with a pair of universal primers, 27 F (5′-AGAGTTTGATCCTGGCTCAG-3′) and 1541R (5′-AAGGAGGTGATCCAGCC GCA-3′). Amplification was performed in a 9700 PCR meter (Bio-Rad Laboratories, Hercules, CA, USA) under the following conditions: 95°C for 5 min; 30 cycles of 94°C for 1 min, 60°C for 30 s; and finally 72°C for 10 min. The PCR products were then purified and cloned into vector pMD19-T using the pMD19-T vector system I kit according to manufacturer instructions (Takara, Dalian, China).

Sequencing was performed by an ABI-Prism model 3730 automated sequencer (Perkin-Elmer, Forest City, CA, USA). The nucleotide sequences were compared with the sequences in the GenBank/EMBL/DDBJ nucleotide sequence databases by the BLASTN program (http://www.ncbi.nlm.nih.gov/BLAST/). These sequences were aligned by the Clustal X program. The phylogenetic relationship was constructed by the neighbor-joining method using MEGA 5.1 from the evolutionary distance data corrected by a two-parameter model developed by Kumar [16], and evaluated by bootstrap resampling [17] with 1,000 replicates.

### Physiological characterization and microscopic observation

Physiological characteristics of isolated strains were identified according to the standard protocol that has been conventionally used in bacterial systematics [18]. The cells were imaged with an optical microscope (Olympus BX51, Japan) and electron microscopes including a scanning electron microscope (JSM-6390, JEOL, Japan), transmission electron microscope (JME-1200EX II, Japan), and an atomic force microscope (DI BioScope, Veeco, USA) according to manufacturer instructions.

### Fermentation tests

The isolated strains were cultivated anaerobically in MA medium. The inoculum acquired after 60 h incubation was added at 10% v/v with 100 mL medium in 250-mL glass serum bottles with the pH adjusted to 7.0. In the test of evaluation for H_2_ production from various substrates, the isolated strains were grown on glucose, xylose, xylan filter paper, microcrystalline cellulose (Avicel PH-101, Fluka), and washed or unwashed lignocellulosic materials (wheat straw, corn cob, and corn stalk), with a fixed concentration of 5 g/L. All reactions were carried out in a water-heating incubator at 150 rpm, 60°C. Samples were taken at predetermined intervals for determination of cell mass, residual carbon substrate concentration, quantity and compositions of produced biogas, pH, and metabolic products in liquid phase.

### Analytical methods

The cell density was determined by measuring the total protein content of the culture using a modified method described by Bradford [10]. Gases were measured using a gas chromatograph (GC) (4890D, Agilent Cooperation, USA) equipped with a thermal conductivity detector (TCD). Volatile fatty acids and alcohols were also detected by a GC (4890D, Agilent Cooperation, USA) equipped with a hydrogen flame-ionization detector (FID) and a 2.0-m stainless steel column packed with GDX103 (60/80 mesh). Cellulosic residual substrates were determined gravimetrically after drying at 80°C for two days with non-inoculated medium as a control. The chemical components and their individual degradation efficiencies were measured according to the methods described by Goering et al. [19]. The corn stalk morphology changeswere examined by scanning electron microscopy (SEM). To recognize the whole-cells structure under SEM, the corn stalk stem was cut into 0.5- to l.0-cm pieces. After 96 h of fermentation, the corn stalk specimens were mounted on stubs and sputter-coated with gold prior to imaging with a JEOL JSM-840 scanning electron microscope using 5-kV accelerating voltage and 10-mm distance. Digital images were captured using 1,280 × 960 resolution and 160-s dwell time. The endoglucanase, exoglucanase (avicelase), cellobiase, and xylanase activities were determined by measuring reducing sugars released from an appropriate substrate according to the method described by Rattanachomsri et al. [20]. In brief, the reaction mixture for the enzyme analysis contained 0.5 mL of enzyme solution or culture supernatant and 1.5 mL of 1.0% corresponding substrate in 0.05 mol/L citrate acid buffer, pH 5.0: carboxymethyl cellulose (CMC) for endoglucanase activity, avicel cellulose for exoglucanase activity, cellobiose for cellobiase activity, and birchwood xylan for xylanase activity. After incubation at 60°C for 60 min, the amount of reducing sugars was determined from the absorbance measurements at 540 nm. One unit of enzyme activity (IU) was defined as the amount of enzyme which produced 1 μmol of reducing sugar per 1 min. All assays were performed in triplicate and the mean was reported with standard deviation.

## Results

### Enrichment of cellulose-degrading thermophilic bacteria for H_2_ production

**Figure 1:**
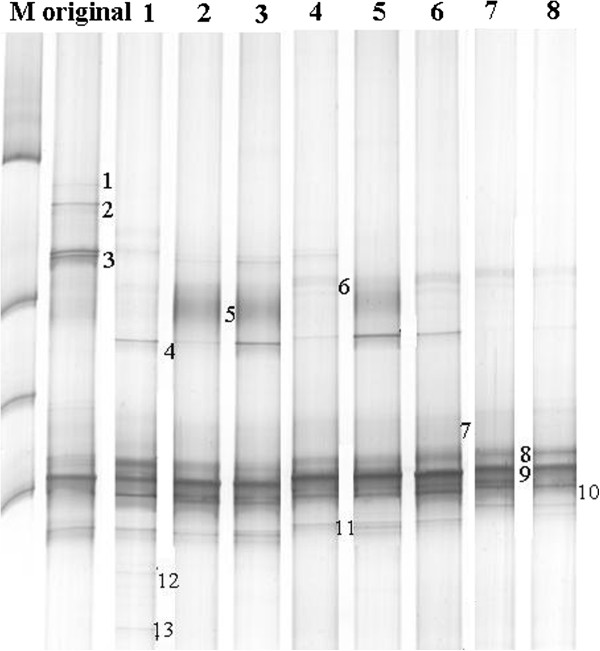
**DGGE profiles based on 16S rDNA from different enrichment cultures.** Bands 1 to 13 were extracted from the gel for sequencing.

For enrichment of cellulolytic bacteria, repeated subcultures were run until stable cellulose-digesting communities were established. Positive growth was determined by an increase in turbidity and production of yellow pigment in the serum bottles containing Avicel as the sole carbon source. From the enrichments, the culture enriched from rooted wood crumb displayed faster decomposition of Avicel and substantial production of hydrogen; thus, it was selected for analyzing the microbial community. As shown in Figure 1, some of the existing bands in the original enrichment disappeared at a later period. However, bands 6, 7, 8, 9, and 10 were all present in the consecutive subcultures. After a seven-generation cultivation, the microbial content indicated by the number and intensity of the bands in the DGGE gels was constant over the next two generations, which demonstrated that the enriched culture after seven transfers had an invariable composition. The major bands in each generation were excised and purified to determine the sequence. After sequencing and BLAST analysis, bands 1, 2, 3, and 13 were identified as uncultured bacteria of *Clostridium* sp. and *Paenibacillus* sp.; bands 4, 5, 11, and 12 were identified as *Clostridium thermocellum*, *Clostridium cellulosi*, *Clostridium caenicola*, and *Thermoanaerobacterium thermosulfurigenes*. However, bands 6, 7, 8, 9, and 10 were all identified as *Thermoanaerobacterium thermosaccharolyticum*, suggesting that the species *T. thermosaccharolyticum* was the fittest survival among the seven-generation enriched cultivation (Table 1). This result implies that the *T. thermosaccharolyticum* group was enriched as dominant in the bacterial community structure of this system and that this organism participates in cellulose hydrolysis.

**Table 1:** Retrieved results of DGGE bands by BLASTn and sequence match for different culture samples

**Bands**	**Most similar sequence (accession number)**	**Identity (%)**
1,2	Uncultured *Clostridium* sp. clone TUM-Mbac-TR1-B1-K2-70(EU812977.1)	99%
3	Uncultured bacterium clone AKIW809 (DQ129400.1)	93%
4	*Clostridium thermocellum* strain CTL-6 (FJ599513.1)	99%
5	*Clostridium cellulosi* (FJ465164.1)	90%
6-10	*Thermoanaerobacterium thermosaccharolyticum* (M59119)	99%
11	*Thermoanaerobacterium thermosulfurigenes* (L09171.1)	96%
12	*Clostridium caenicola* (AB221372.2)	97%
13	Uncultured *Paenibacillus* sp. JAB SHC 24 (AY694512.1)	95%

### Isolation and characterization of pure cultures

**Figure 2:**
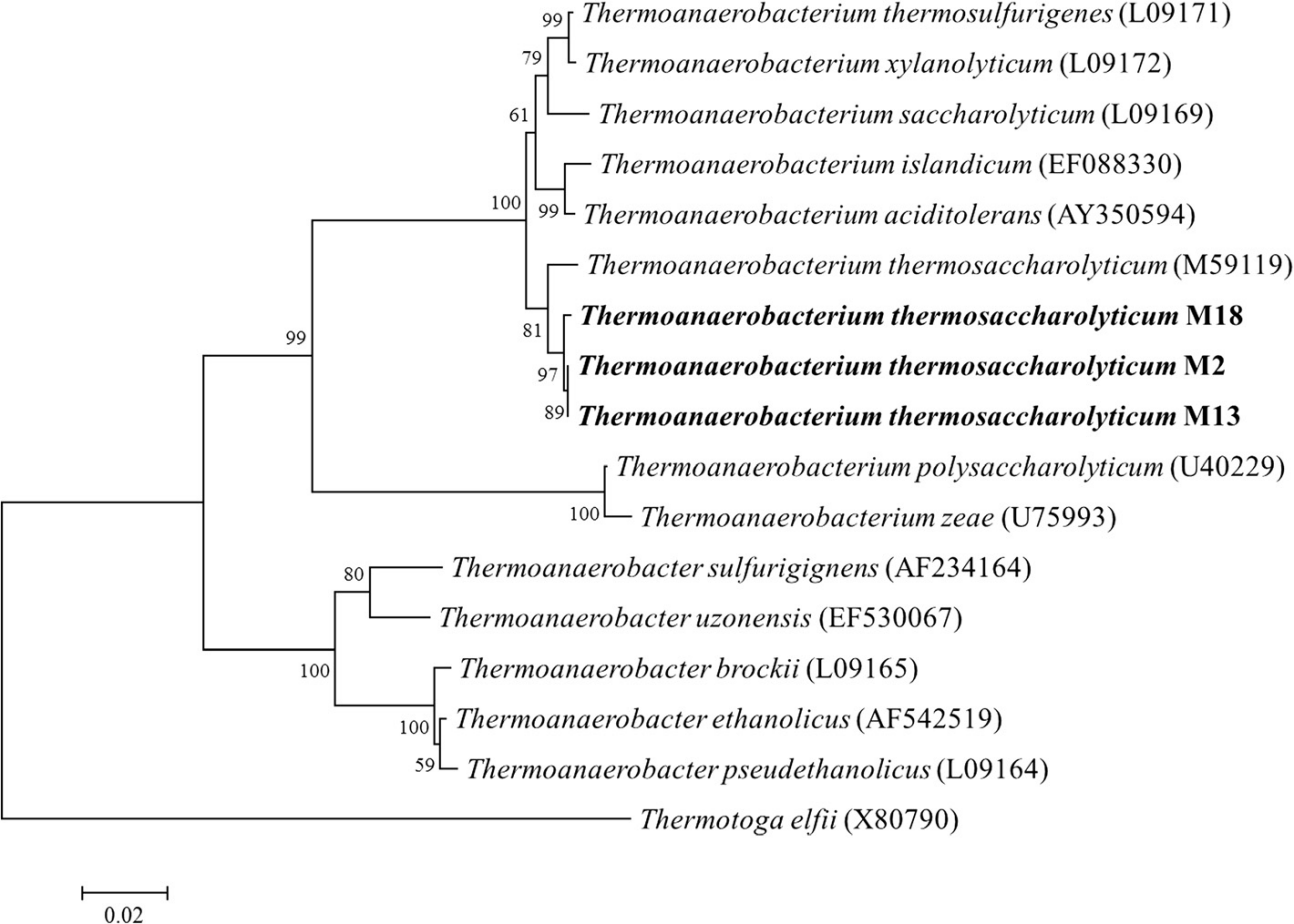
**Phylogenetic tree between isolated strains and related species based on 16S rRNA gene sequences.** Bar represents 2% sequence divergence.

In order to purify the above-described organisms, the serially diluted active and stable enriched culture was plated on cellulose agar, and the bacterial colonies with extensive clearing zones were screened. Three strains of M2 (GenBank accession number KJ162237), M13 (GenBank accession number KJ162236), and M18 (GenBank accession number FJ465165), capable of producing hydrogen from cellulose, were isolated from the enrichment culture. Based on the similarity analysis of the 16S rRNA gene sequence, all isolated strains belonged to the genus *Thermoanaerobacterium*, and the closest relationship was with *Thermoanaerobacterium thermosaccharolyticum* DSM571 (formerly *Clostridium thermosaccharolyticum*) at a similarity ranging from 97.6% to 99.8% (Figure 2), suggesting that the dominant functional bacterium presented in the enrichment cultures was successfully isolated.

**Figure 3:**
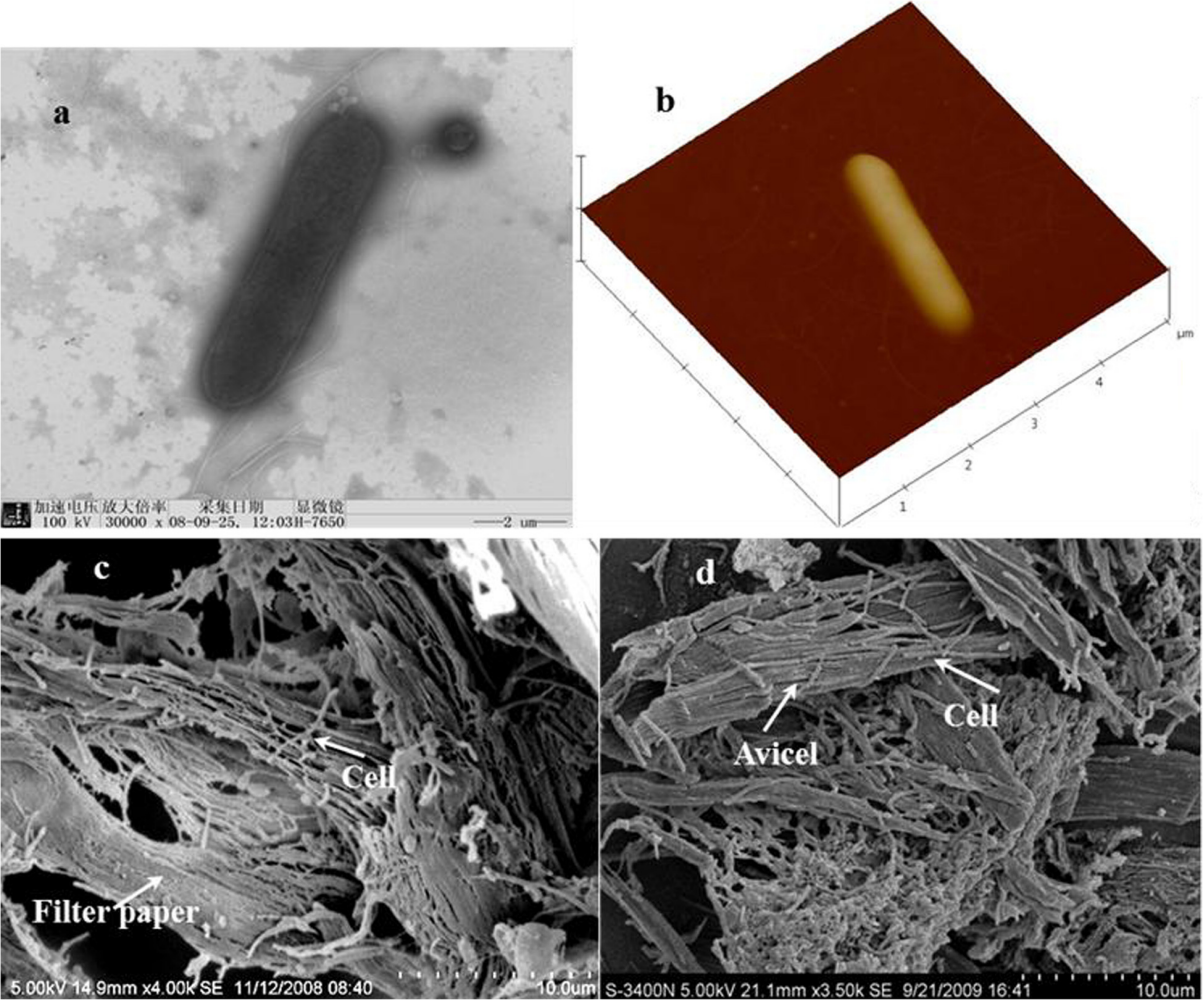
**Micrographs of *T. thermosaccharolyticum *M18. (a)** Transmission electron microscopy (TEM) micrograph, **(b)** atomic force microscopy (AFM) micrograph, and scanning electron microscopy (SEM) images of strain M18 cells associated with the cellulose fibers supplied in the filter paper culture **(c)** and Avicel culture **(d)**.

The physiological properties of the isolated strains were similar to those that have been previously described for *T. thermosaccharolyticum*[21-24]. They were rod-shaped (0.3 μm to 0.8 μm × 1.5 μm to 5 μm) with rounded ends, and flagella were also observed (Figure 3a,b). It was noted that the strains varied their morphologies with the carbon sources used; the cells grown on cellulose were 2 to 10 times longer than the cells grown on glucose, xylose, and xylan (Figure 3c,d). All cultures grown on Avicel produced a light yellow pigment or an orange yellow pigment.

### Fermentation on defined carbohydrates

Enrichment results suggest that *T. thermosaccharolyticum* was the dominant species during transfers and displayed activity in cellulose hydrolysis. Further, to assess the performance, the isolated strains were cultured in MA medium supplied with various defined plant polymers, including microcrystalline cellulose (Avicel), filter paper, xylan, xylose, and glucose. As shown in Table 2, all strains displayed effective utilization on monosaccharides of glucose and xylose, which resulted in complete degradation of these substrates within 24 h. This confirms previous results obtained showing the ability of *T. thermosaccharolyticum* to utilize pentose and hexose [24]. Xylan also supported intensive growth of these strains. The degradation of xylan occurred immediately, even though the inoculum was obtained on a medium with cellulose. At the end of fermentation, nearly all the xylan was used up. Compared to the use of xylan as a substrate, considerable amounts of H_2_ were acquired on Avicel and filter paper, but its fermentation was slow and incomplete. After three days fermentation, the highest hydrogen production was observed for M18 with a cellulose degradation of 81.5%, whereas the other strains gave a moderate cellulose degradation range of 47.5% to 72.2%. In all substrates, the major fermentation products were acetate and butyrate with small amounts of ethanol, propionate, and butanol.

**Table 2:** Fermentation products of cellulolytic strains isolated from enrichment culture

**Strain**	**Substrate (5 g/L)**	**Degradation (%)**	**Fermentation products (mmol/L)**					
			**Ethanol**	**Butanol**	**Acetate**	**Propionate**	**Butyrate**	**H**_**2**_
M2	Glucose	100 ± 0.29	6.70 ± 0.20	0.87 ± 0.09	25.8 ± 1.02	2.55 ± 0.29	13.7 ± 0.24	54.9 ± 0.89
	Xylose	100 ± 0.07	5.55 ± 0.46	0.88 ± 0.09	24.2 ± 0.21	1.72 ± 0.21	12.4 ± 0.77	55.7 ± 0.25
	Xylan	92.1 ± 0.64	5.06 ± 0.26	0.75 ± 0.10	21.6 ± 0.31	1.64 ± 0.15	11.0 ± 0.33	43.2 ± 0.22
	Filter paper	71.2 ± 0.24	5.46 ± 0.52	3.25 ± 0.30	18.1 ± 0.20	2.65 ± 0.34	8.89 ± 0.12	35.5 ± 0.20
	Cellulose	67.1 ± 2.21	4.78 ± 0.21	1.51 ± 0.18	17.1 ± 0.79	1.53 ± 0.32	9.08 ± 0.20	38.5 ± 0.76
M13	Glucose	100 ± 0.20	4.18 ± 0.23	0.87 ± 0.04	22.8 ± 0.24	1.55 ± 0.20	13.2 ± 0.18	55.4 ± 0.23
	Xylose	100 ± 0.43	5.07 ± 0.12	0.75 ± 0.07	23.3 ± 0.27	1.32 ± 0.06	11.8 ± 0.35	54.6 ± 0.04
	Xylan	87.5 ± 1.28	5.13 ± 0.18	0.97 ± 0.06	21.9 ± 0.22	1.16 ± 0.12	10.2 ± 0.09	40.2 ± 0.42
	Filter paper	54.3 ± 0.83	3.76 ± 0.24	1.02 ± 0.21	10.1 ± 0.25	0.89 ± 0.04	4.89 ± 0.20	24.9 ± 0.81
	Cellulose	46.5 ± 2.12	2.06 ± 0.12	0.50 ± 0.02	9.81 ± 0.13	0.76 ± 0.02	5.12 ± 0.10	20.8 ± 2.25
M18	Glucose	100 ± 0.00	6.04 ± 0.10	0.95 ± 0.09	24.8 ± 1.15	1.05 ± 0.16	14.2 ± 0.22	56.5 ± 0.53
	Xylose	100 ± 0.04	6.55 ± 0.55	2.10 ± 0.07	24.1 ± 1.41	1.32 ± 0.02	12.8 ± 0.89	57.2 ± 1.33
	Xylan	100 ± 1.24	5.06 ± 0.85	0.75 ± 0.08	23.6 ± 0.44	1.14 ± 0.10	13.2 ± 0.45	54.0 ± 0.42
	Filter paper	80.8 ± 0.24	5.76 ± 0.45	4.43 ± 0.37	20.1 ± 0.84	2.65 ± 0.22	10.8 ± 0.24	42.5 ± 1.11
	Cellulose	81.5 ± 0.24	5.14 ± 0.25	2.34 ± 0.09	22.6 ± 0.88	1.15 ± 0.02	12.6 ± 0.33	43.8 ± 0.94

**Figure 4:**
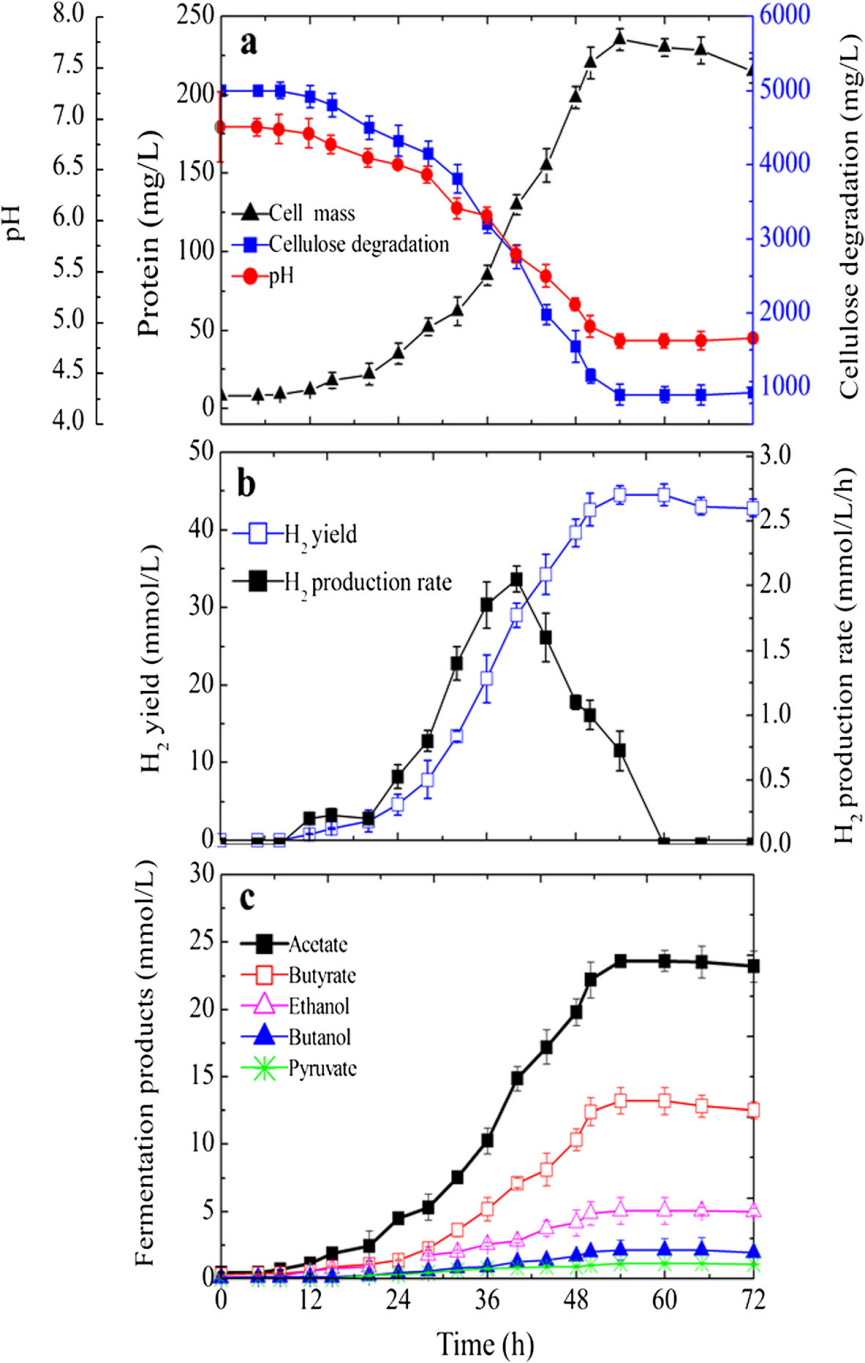
**The time course profiles and kinetics of batch fermentation in 5 g/L Avicel medium.** Data points are the means of triplicate cultures with the error bars indicating standard deviations.

Based on the robust anaerobic growth and efficiency of hydrogen production from various carbohydrates, the intriguing strain M18 was further investigated for its dynamic of cellulose fermentation with 5 g/L Avicel. As shown in Figure 4a, exponential cell growth was observed after a 15-h lag phase, and the cell mass reached a maximum of 225 mg/L at 54 h. Coinciding with the cell growth, the cellulose degradation did not occur before 15 h; it then promptly hydrolyzed during the fermentation at 20 to 48 h. This observed lag phase for cellulose degradation can be attributed to the synthesis and assembly of a multi-enzyme complex [25]. At the onset of the stationary phase, the cellulose was not completely consumed, which might be caused by the depletion of a particular nutrient from the culture medium [26] or the inhibitory intracellular compounds accumulated in the cells resulting from an inefficiently regulated carbon flow [27]. The pH gradually decreased throughout the fermentation and reached a final value of 5.02, which also potentially contributed to the limitation of cellulose degradation, as other studies have shown that a pH lower than 5.5 leads to no cellulose degradation. To address this limitation, running reactors in continuous mode and increasing the buffer capacity were recommended as alternative strategies to improve the extent of cellulose degradation.

As the degradation of Avicel proceeded, H_2_ and liquid products were produced and accumulated as shown in Figure 4b,c. H_2_ production started from the early exponential phase (4 h), and the rate of H_2_ production reached a maximum in the late exponential phase. At the end of fermentation, the cumulative hydrogen production was estimated to be 44.5 mmol/L with a corresponding yield of 10.9 mmol/g Avicel consumed. Gas chromatography (GC) analysis showed that the end liquid products were primarily composed of acetate and butyrate with a molar ratio (acetate/butyrate) near 1.8.

### Fermentation on untreated lignocellulosic substrates

**Figure 5:**
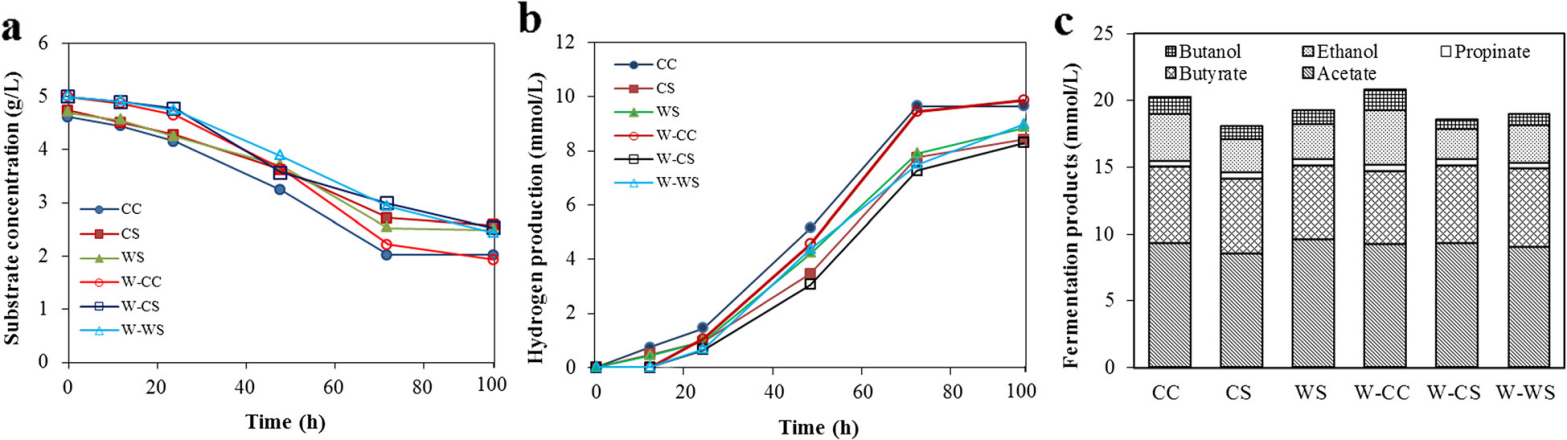
**Profiles of lignocellulosic materials for hydrogen production. (a)** Residual weight of substrates, **(b)** amount of hydrogen produced, and **(c)** liquid fermentation end products.

Using natural lignocellulosic biomasss as a feedstock for biofuel production usually requires expensive and time-consuming pretreatment processes to facilitate making the insoluble carbohydrates more accessible to hydrolytic enzymes. However, economic analyses have revealed that about 20% of the projected costs is ascribed to the pretreatment [9]. The ability to utilize untreated lignocellulosic material was investigated here with *T. thermosaccharolyticum* M18. Three different polymeric substrates, corn cob (CC), corn stalk (CS), and wheat straw (WS), were examined at 0.5% (w/v). To demonstrate the contribution of insoluble substrates rather than soluble extractives released by autoclaving for untreated biomass on hydrogen production, after autoclaving all materials were washed extensively with hot water and the washed residues together with the control without wash were used as the sole carbon source for the cultivation of strain M18 (Figure 5). It was found that strain M18 grew well on all these substrates, and there was no significant difference in hydrogen production for the washed and unwashed materials. It should be noted that H_2_ was detected after 10 to 15 h incubation for the unwashed samples, while no H_2_ was measured until 24 to 30 h incubation for the washed samples. As expected, approximately 5% of the tested substrates were solubilized by autoclaving, which was quickly consumed and responsible for producing H_2_ during the start-up period in the case of the unwashed materials (Figure 5a). But the subsequent H_2_ production from the unwashed substrates became slow and showed a similar trend to that of the washed substrates. These results indicated that the soluble extractives released from the raw materials had no negative effect on the growth of strain M18. Moreover, strain M18 was able to utilize insoluble carbohydrates present in raw lignocellulosic materials as well as Avicel as sources of carbon and energy for H_2_ production. The maximum weight loss took place within 96 h, and the weight losses were 2.98 ± 0.10, 2.42 ± 0.04, and 2.51 ± 0.22 g, corresponding to a utilization of 62.71%, 56.07%, and 59.23% of insoluble carbohydrates in CC, CS, and WS, respectively (Table 3). In all cases, the cumulative hydrogen production reached its highest value after 72 to 96 h inoculation (Figure 5b). Consistent with the degradation of insoluble carbohydrates, a larger amount of hydrogen was produced on CC than on the other alternative substrates. The rate of production of H_2_ was similar for CS and WS, but somewhat lower than for CC. In general, comparable yields of hydrogen were calculated for all three substrates. Similar to the growth on defined substrates, volatile fatty acids were the major end products, acetate and butyrate accounting for more than 80% of the volatile fatty acid total (Figure 5c).

**Table 3:** **H**_**2 **_**production by *T. thermosaccharolyticum *** **M18 on polymeric substrates after 96 h fermentation**

**Parameters**	**Lignocellulosic substrates**					
	**Corn stalk**		**Wheat straw**		**Corn cob**	
	**Unwashed**	**Washed**	**Unwashed**	**Washed**	**Unwashed**	**Washed**
Initial substrates (g/L)	5.0 ± 0.02	5.0 ± 0.01	5.0 ± 0.01	5.0 ± 0.02	5.0 ± 0.03	5.0 ± 0.00
Residual substrate (g/L)	2.58 ± 0.05	2.47 ± 0.14	2.49 ± 0.22	2.43 ± 0.28	2.02 ± 0.10	1.93 ± 0.14
Insoluble carbohydrates utilized (%)	56.07 ± 1.46	57.12 ± 2.18	59.23 ± 2.56	52.07 ± 2.43	62.71 ± 3.11	58.88 ± 2.72
Maximum H_2_ production (mmol/L)	8.42 ± 1.42	8.30 ± 0.88	8.86 ± 0.44	8.98 ± 0.32	9.65 ± 1.48	9.88 ± 0.76
H_2_ production rate (mmol/L/h)	0.10 ± 0.02	0.09 ± 0.02	0.11 ± 0.02	0.11 ± 0.02	0.13 ± 0.03	0.11 ± 0.02
H_2_ yield (mmol/g substrate)	3.47 ± 0.11	3.28 ± 0.33	3.53 ± 0.24	3.49 ± 0.18	3.23 ± 0.30	3.27 ± 0.21

### Biodegradation characteristics of lignocellulosic biomass

**Figure 6:**
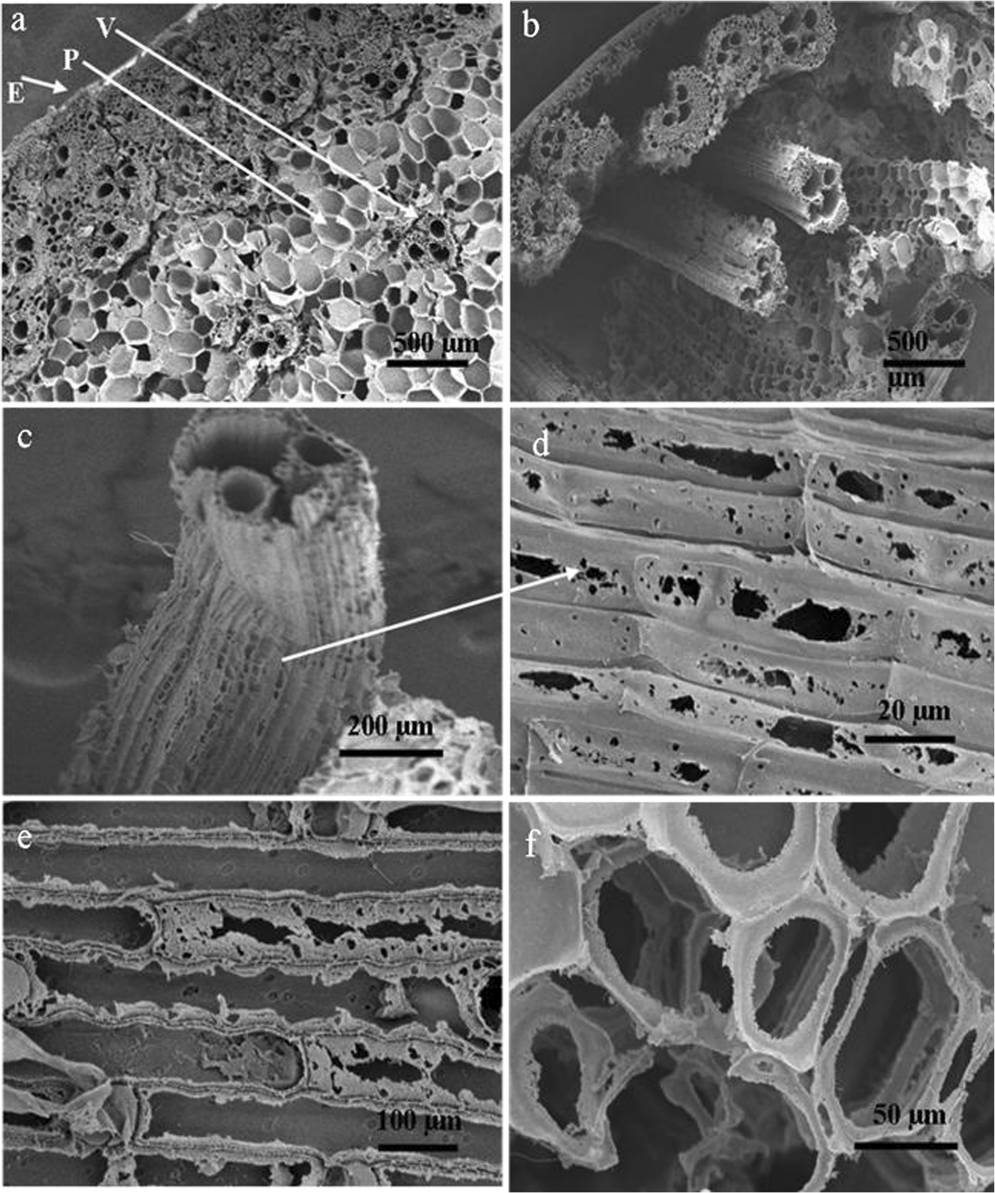
**SEM images of corn stalk stem samples: unfermented corn stalk stem (a); corn stalk stem after fermentation (b); vascular bundle cells after fermentation (c, d); epidermis cells after fermentation (e); parenchyma cells after fermentation (f).** E: epidermis; P: parenchyma; V: vascular bundles.

**Figure 7:**
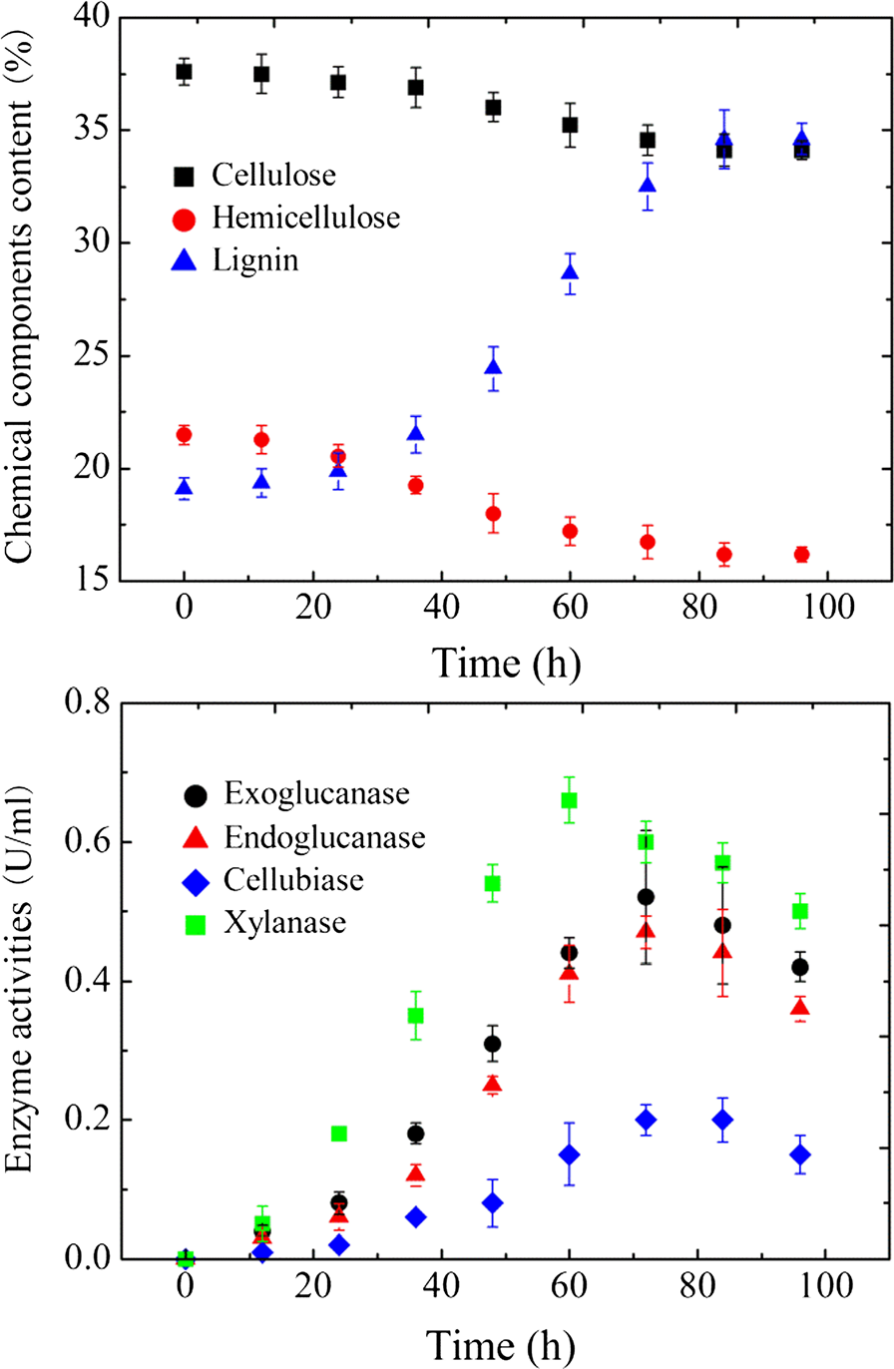
**Chemical components content of corn stalk and relative enzyme activities at different incubation times with M18.** Data points are the means of triplicate cultures with the error bars indicating standard deviations.

To elucidate the biodegradation characteristics of M18 on lignocellulosic biomass, CS was taken as a representative substrate. The morphology changes induced by incubation with M18 were examined by SEM to provide direct insight into the structure modification in the CS. Before fermentation, various types of ordered cell walls were easily recognized in the unfermented CS sample, including epidermis cells, parenchyma cells, vascular bundles (phloem and xylem), and thick-walled fiber cells (Figure 6a). After fermentation by M18, the residual CS was changed dramatically; the initial connected structure was destroyed and separated and was subsequently replaced by a collapsed and distorted cell wall structure (Figure 6b). Clearly, the basic cell wall components were greatly changed in appearance during fermentation. Specifically, caves and fractures appeared on the vascular bundle surface shown in a vertical-sectional view (Figure 6d), and specific zones of the phloem tissue corresponding to the cells located between vessels disappeared from the cross-sectional view (Figure 6c). The structure of the epidermis cells was also destroyed after fermentation; the cutical waxy layer appeared to be almost desquamated, and the microfibrils were exposed to the surface. Pits and cracks could be observed on the underlying tissue after dewaxing (Figure 6d). In particular, the parenchyma cells consisting of polygons at regular ranges suffered the most serious damage, in which clear ringed cavities were formed or some cells were completely degraded (Figure 6e). These unique cells are largely unlignified and are composed of cellulose and hemicellulose [28].In addition to the results from SEM observation, the changes of the chemical components and corresponding hydrolysis enzyme during fermentation were also analyzed. As shown in Figure 7, the cellulose and hemicellulose components were gradually degraded. The content of hemicellulose decreased quickly within the first 48-h fermentation, whereas the cellulose content dropped rapidly in the following fermentation time of 48 to 72 h. At the end of fermentation, the dry weight loss values of cellulose and hemicellulose were 48.8% and 55.6%, respectively. Consistent with the apparent weight loss of cellulose and hemicellulose, the xylanase activity increased rapidly before 48 h and reached a maximum level after 60 h of incubation at 0.66 U/mL, but the endoglucanase, exoglucanase (avicelase), and cellobiase activities were not notably detected until 48 h thereafter, achieving maximum activities of 0.51 U/mL, 0.48 U/mL, and 0.16 U/mL. These observations further confirmed the degradation of cellulose and hemicellulose from untreated lignocellulose complex by strain M18.

### Fermentation with single culture versus co-cultures

Previous studies used the co-culture of cellulolytic strains and hydrogen producers by taking advantage of their specific metabolic capacities to improve the conversion efficiency of cellulosic substrates to hydrogen [29]. This work tested whether the newly isolated cellulolytic strain M18 can work with other strains for bioaugmented biohydrogen production from Avicel and lignocellulosic substrates. A well-characterized strain, *T. thermosaccharolyticum* W16, which can produce hydrogen efficiently from glucose and xylose [24], and the newly isolated strains M2 and M13 were employed to establish defined dual co-cultures with M18. All of the two-strain combinations were mixed at the same volumes in co-cultures. Interestingly, although the co-culture M18 and M13 produced a little more hydrogen than the single culture after 96 h, the hydrogen production in the other co-cultures on different substrates was comparable to that obtained by single strain M18 (Table 4). Therefore, the tested co-culture fermentation of cellulose and CS had no beneficial effect on hydrogen production versus the single strain.

**Table 4:** **Fermentation products of *T. thermosaccharolyticum *** **M18 in co-cultures with M2, M13, and W16**

**Culture**	**Substrate**	**Degradation**	**Fermentation products (mmol/L)**					
	**(5 g/L)**	**(%)**	**Ethanol**	**Butanol**	**Acetate**	**Propionate**	**Butyrate**	**H**_**2**_
M18 + M2	Avicel	84.54 ± 1.23	5.15 ± 0.21	1.97 ± 0.07	24.83 ± 1.46	1.05 ± 2.29	14.08 ± 0.55	46.7 ± 0.07
M18 + M13		81.50 ± 0.24	4.87 ± 0.30	2.33 ± 0.14	22.76 ± 0.49	0.97 ± 0.05	13.10 ± 0.23	43.8 ± 1.19
M18 + W16		80.45 ± 0.68	5.01 ± 0.43	2.09 ± 0.09	23.44 ± 1.48	1.34 ± 0.10	12.98 ± 0.57	43.8 ± 0.87
M18 + M2	Corn stalk	52.31 ± 1.82	2.45 ± 0.43	0.77 ± 0.10	10.21 ± 0.24	0.56 ± 0.04	5.41 ± 0.12	8.98 ± 0.21
M18 + M13		49.86 ± 2.09	2.15 ± 0.20	0.80 ± 0.11	8.97 ± 0.42	0.52 ± 0.05	6.09 ± 0.35	8.56 ± 0.18
M18 + W16		48.42 ± 1.56	1.97 ± 0.23	0.78 ± 0.06	9.12 ± 0.46	0.49 ± 0.09	5.18 ± 0.24	8.46 ± 0.13

### Fermentation balances

The preceding data showed that *T. thermosaccharolyticum* M18 had a great potential for hydrogen production directly from cellulosic biomass. However, an understanding of the fermentation process is a prerequisite for this strain to achieve a high product yield in an applied context. In this respect, high quality performance data validated by carbon mass balance is desired. The carbon balances for *T. thermosaccharolyticum* M18 grown to stationary phase accounted for 96.9% and 106.8% of the carbon initially present in the substrate for Avicel and CS, respectively (Table 5). These values revealed carbon recoveries close to 100%. The distribution of carbon among all constitutes demonstrated that acetate and butyrate were the major end products for both Avicel and CS fermentation, whereas no lactate was determined. In contrast, lactate was the main end product of *C. thermocellum* grown on cellulose and CS [29], indicating that the carbon metabolism of *T. thermosaccharolyticum* M18 was significantly different from that of *C. thermocellum*. On the other hand, no sugars were detected in the culture grown on Avicel and CS during the stationary period. In comparison, glucose, xylose, and cellobiose were accumulated for *C. thermocellum* in the culture broth supplemented with cellulose and CS during anaerobic fermentation.

**Table 5:** **Fermentation balances of carbohydrates by *T. thermosaccharolyticum *** **M18 on Avicel and corn stalk**

**Culture**	**Substrate**	**Substrate initial (g/L)**	**Substrates consumed (g/L)**	**Ethanol (mM)**	**Acetate (mM)**	**Propionate (mM)**	**Butanol (mM)**	**Butyrate (mM)**	**CO**_**2 **_**(mM)**	**Cell mass (mM)**	**Carbon recovery (%)**
M18	Avicel	5.0	4.08 ± 0.12	5.04 ± 0.25	23.61 ± 0.05	1.13 ± 0.02	2.10 ± 0.07	13.21 ± 0.33	24.62 ± 1.01	9.95 ± 0.13	96.9 ± 1.87
	Corn stalk	5.0	2.42 ± 0.08	2.23 ± 0.41	9.51 ± 0.41	0.50 ± 0.01	0.84 ± 0.14	5.50 ± 0.65	8.67 ± 0.19	7.17 ± 0.08	106.8 ± 2.25

## Discussion

CBP offers great potential for lower cost and higher efficiency compared to processes featuring dedicated cellulase production [29,30]. However, the desired microorganisms for cellulosic material conversion to biofuel via CBP are not currently available. In the present work, the objective was to isolate thermophilic bacteria suitable for a single-step conversion of lignocellulosic biomass to hydrogen. To determine whether the representative microorganisms present in the enrichment were subsequently isolated, the microbial community profile during enrichment was monitored by DGGE. Fortunately, after selective cultures growing at 60°C for enrichment of cellulose-degrading microorganisms, three cellulolytic strains of the genus *Thermoanaerobacterium* were isolated, indicating that the functional strains of interest were successfully cultured from enrichment. All isolates were able to ferment microcrystalline cellulose, filter paper, and xylan, as well as glucose and xylose. However, no study has demonstrated that the strains in genus *Thermoanaerobacterium* could be used as the sole microorganism to accomplish both cellulose degradation and H_2_ generation, even though many reports have illustrated that several species of genus *Thermoanaerobacterium* possess the capability to utilize various macromolecules accompanied by H_2_ production [21-24,31,32], including *T. thermosaccharolyticum*, *T. polysaccharolyticum*, *T. zeae, T. lactoethylicum*, *T. aotearoense*, and *T. saccharolyticum*. This result appears to indicate the presence of a cellulolytic characteristic in *Thermoanaerobacterium* sp., which thus represents a novel cellulolytic bacterium distinguished from all other known cellulolytic bacteria.

Among the isolated strains, M18 exhibited a higher capability of hydrogen production from tested carbohydrates, making it more suitable for the hydrolysis and fermentation of cellulosic substrates. When *T. thermosaccharolyticum* M18 was grown on microcrystalline cellulose, more than 80% of Avicel was utilized with a maximum H_2_ production of 44.5 mmol/L, yielding 10.9 mmol H_2_/g Avicel consumed. The high hydrogen production from cellulose reported here was obtained with the strain *T. thermosaccharolyticum* M18 grown on non-optimized medium under non-optimized cultivation conditions. This value is as much or more than the H_2_ levels reported for the thermophilic cellulolytic bacterium *C. thermocellum*, an extensively studied CBP candidate. When *C. thermocellum* JN4 was co-cultured with *T. thermosaccharolyticum* GD17, similar yield of H_2_ to that of M18 was achieved on cellulose fermentation. However, less than one-third of the H_2_ yield of M18 was obtained when a single culture of *C. thermocellum* JN4 was employed, indicating that the co-culture for *C. thermocellum* resulted in a high H_2_ yield. In contrast, the single strain M18 performed as efficiently as the co-culture of *C. thermocellum* JN4 and *T. thermosaccharolyticum* GD17 [12].

The cellulolytic strain *T. thermosaccharolyticum* M18 grew well on insoluble carbohydrates (mainly hemicellulose and cellulose) contained in untreated lignocellulosic substrates. At 5 g/L substrate concentration, *T. thermosaccharolyticum* M18 utilized 56.07% to 62.71% of insoluble carbohydrates in untreated corn cob, corn stalk, and wheat straw. All of the alternative lignocellulosic substrates produced similar final yields of H_2_. In comparison, *C. thermocellum* ATCC 27405 produced 1.07 mmol H_2_/g substrate on both dried distiller grains (DDGs) and barley hulls (BH), a co-culture of *C. thermocellum* JN4 and *T. thermosaccharolyticum* GD17 produced 1.83 mmol H_2_/g wheat straw, and an extremely thermophilic *Caldicellulosiruptor saccharolyticus* produced H_2_ from wheat straw, sweet sorghum plant, maize leaves, and bagasse with yields varying from 0.67 to 1.83 mmol/g substrate [12,13,33]. Compared to the data of the present study (3.25 to 3.53 mmol H_2_/g substrates), this suggests that *T. thermosaccharolyticum* M18 could produce about a two to five times higher H_2_ yield on lignocellulosic substrates.

Biodegradation characteristics from SEM observation further confirmed the degradation of insoluble cellulose and hemicellulose from lignocellulose complex. The ability of *T. thermosaccharolyticum* M18 to utilize major carbohydrates of hemicellulose and cellulose can be attributed to the presence of a large set of cellulase and xylanase. During hydrolysis of corn stalk, the degradation of hemicellulose declined quickly within the first 48-h fermentation, whereas cellulose degradation was not apparent until 48 h. One could assume that *T. thermosaccharolyticum* M18 preferentially utilizes hemicellulose, where the hemicellulose degradation on the one hand increases the biomass, and on the other hand reduces the steric hindrance of hemicellulose, thus exposing more cellulose to the bacteria, which can speed up the cellulose degradation. Considering that *C. thermocellum* cannot metabolize hemicellulose [34], the active utilization of both cellulose and hemicellulose for *T. thermosaccharolyticum* M18 makes it more attractive. Overall, the results presented here demonstrate that *T. thermosaccharolyticum* M18 could be a promising candidate for converting lignocellulosic feedstock in a single step to H_2._

Although the amounts of hydrogen produced by the novel cellulolytic *T. thermosaccharolyticum* M18 are relatively high compared to other studies, increasing the H_2_ production efficiency from lignocellulosic materials is still an essential issue for establishing an applicable platform for converting lignocellulose to H_2_. In this respect, optimization of culture conditions in terms of physicochemical parameters such as substrate loading, pH, and temperature, targeted metabolic modification with multiple transgenes, and integrated processes including a coupled system of dark and photo-fermentative H_2_ production and a system of dark fermentation coupled with bioelectrohydrogenesis will be addressed carefully in the future.

## Conclusions

This study investigates the use of a newly isolated, moderately thermophilic bacterium, M18, to produce HB_2_ directly from lignocellulosic materials. The bacterium was isolated from rotten wood crumb and identified as *Thermoanaerobacterium thermosaccharolyticum* and is designated here as *T. thermosaccharolyticum* M18. This is the first report of a *Thermoanaerobacterium* genus capable of producing H_2_ directly from various pure and natural cellulosic substrates such as filter paper and corn stalk via acetate-butyrate-type fermentation. Overall, strain M18 can rapidly produce high yields of H_2_ directly from lignocellulosic materials, and thus could be a promising candidate for lignocellulose bioconversion processes.

## Abbreviations

AFM: atomic force microscopy; Avicel: microcrystalline cellulose; BLAST: basic local alignment search tool; CBP: consolidated bioprocessing; CC: corn cob; CMC: carboxymethyl cellulose; CS: corn stalk; DGGE: denaturing gradient gel electrophoresis; GC: gas chromatograph; HPLC: high performance liquid chromatography; MA: modified ATCC 1191 medium; PCR: polymerase chain reaction; SEM: scanning electron microscope; SHF: separate hydrolysis and fermentation; SSF: simultaneous saccharification and fermentation; TEM: transmission electron microscopy; WS: wheat straw.

## Competing interests

The authors declare that they have no competing interests.

## Authors’ contributions

GLC performed the experiments, analyzed the data, and drafted the manuscript; LZ and ZYW helped with the experimental work. AJW and NQR wrote and revised the manuscript. All authors read and approved the final manuscript.
